# Acute foot and ankle injuries and time return to sport

**DOI:** 10.1051/sicotj/2021024

**Published:** 2021-04-15

**Authors:** Gowreeson Thevendran, Anish R. Kadakia, Eric Giza, Daniel Haverkamp, Jonkheer Pieter D’Hooghe, Andrea Veljkovic, Nasef Mohamed Nasef Abdelatif

**Affiliations:** 1 Consultant Orthopaedic Surgeon, Mount Elizabeth Novena Hospital 38 Irrawaddy Road 329563 Singapore; 2 Professor of Orthopedic Surgery, Northwestern Memorial Hospital Chicago 60611 IL USA; 3 UC Davis Sacremento 95616 CA USA; 4 Xpert Clinics Orthopedics Laarderhoogtweg 12 1101 EA Amsterdam The Netherlands; 5 Department of Orthopaedic Surgery, Aspetar Orthopaedic and Sports Medicine Hospital 29222 Doha Qatar; 6 Department of Orthopaedic Surgery, St. Paul’s Hospital, Footbridge Clinic, University of British Columbia Vancouver V6T 1Z4 BC Canada; 7 Consultant Foot and Ankle Orthopedic Surgeon, Dr. Nasef OrthoClinic Medical Center 11617 Cairo Egypt

**Keywords:** Return to play, Acute foot and ankle injuries, Rehabilitation

## Abstract

Foot and ankle sports injuries encompass a wide spectrum of conditions from simple contusions or sprains that resolve within days to more severe injuries that change the trajectory of an athlete’s sporting career. If missed, severe injuries could lead to prolonged absence from the sport and therefore a catastrophic impact on future performance. In this article, we discuss the presentation of the commonest foot and ankle sports injuries and share recent evidence to support an accurate diagnosis and best management practice.

## Introduction

The foot and ankle are a common site for acute sporting injuries in athletes and recreationally active individuals. Athletes may be severely debilitated with their inability to run, jump, and change directions. The spectrum of injuries can vary from simple sprains to career-threatening injuries in professional athletes. Thus, an accurate diagnosis and early treatment followed by aggressive rehabilitation remains the sine qua non of such injuries.

**Table 1:** Studies comparing Deltoid Ligament repair versus nonrepair in syndesmotic stabilization.

Article	N (repaired DL/unrepaired)	Level of evidence	Outcome	Medial clear space
Gu et al. [22]	40 (20/20)	II	Repair group better AFOAS and VAS pain	Better in DL repair group
Woo et al. [23]	78 (41/37)	III	AOFAS and VAS pain comparable	Better in DL repair group
Zhao et al. [24]	74 (20/54)	III	AOFAS and VAS pain comparable	Better in DL repair group

In this review article, we discuss five common acute foot and ankle injuries and their impact on-time return to sport. Further, we discuss the latest updates in their management and outcomes.

## Lisfranc injuries

Lisfranc injuries can be a devastating injury to both the athlete and non-athletes alike. In the athletic population, minor loss of stability of the midfoot will compromise the high level of function that is demanded from the lower extremity. The most critical aspect of treatment is the identification of the injury and severity of the ligamentous/articular damage. Lisfranc injuries represent a significant compromise to the mechanical stability of the midfoot. The most critical aspect in dealing with these injuries is the identification of the injury as opposed to the nuances of the various surgical options. Nunley and Vertullo Stage I injuries do not require surgical stabilization. However, a period of immobilization and restricted weight bearing is required [1]. Nunley Stage II injuries require surgical intervention in order to restore stability to the midfoot. In these cases, by definition, there is no sagittal instability and surgical treatment needs to only restore coronal stability. Nunley type 3 injuries are more high energy and typically involve the 1st, 2nd, and 3rd TMT joints and in some cases the 4th and 5th as well. When the diagnosis is in doubt, specifically to determine if the patient has a Nunley and Vertullo Stage I vs. II an MRI is a useful tool to determine the integrity of the Lisfranc ligament. In the setting of clear instability of the Lisfranc joint complex, a CT scan is obtained to evaluate for subtle articular comminution that would lead one to pursue an arthrodesis vs. ORIF Both sagittal and coronal instability is present in this situation with a higher failure rate of ORIF in these cases, with a fusion of the midfoot recommended. Persistent midfoot instability will negatively impact the ability of an athlete to play at the elite level. We believe in the concept of minimizing surgical risk with a focus on middle column stability and preservation of medial column mobility.

When counseling both the professional and recreational athlete, it is critical to inform them that some patients will be unable to return to high-level sports, as setting appropriate expectations is critical in this high-demand patient population. Additionally, professional players should be informed about the potential effect of the injury on their performance upon return to play. A Lisfranc injury does not equivocally mean the end of an athletic career as long as the injury is recognized and midfoot stability is restored.

## Turf toe injuries

Ligamentous injuries to the hallux MTP complex commonly referred to as “turf toe injuries,” are common among competitive and recreational athletes. Management goals for these injuries include aggressive rehabilitation and early return to competitive activity without compromising appropriate tissue healing or long-term functional outcomes.

The term “turf toe” generally refers to a hyperextension injury to the hallux MTP joint complex. The injury classically occurs when an axial load is delivered to the heel with the ankle in plantar flexion and the hallux in dorsiflexion [2].

Turf toe injuries are most commonly graded as stretching, partial tearing, and complete tearing injuries. These are designated as grades 1, 2, and 3 respectively and objectively represent the spectrum of injury. The physical examination should include the following: Evaluation for patterns of swelling and ecchymosis, stability of MTP joint with dorsal drawer, and varus/valgus testing. Standard weight-bearing AP, lateral and sesamoid axial views should be obtained initially. Comparison to the contralateral side will allow for evaluation of the sesamoid position and possible retraction [3].

Additional studies can include a static forced dorsiflexion view of the first MTP joint or if possible dynamic fluoroscopic evaluation: a ruptured plantar plate will result in the inability of the sesamoids to track with the proximal phalanx. Magnetic resonance imaging (MRI) T2 weighted images will demonstrate soft tissue disruption and can be particularly helpful for partial injuries [4].

Grade 1 turf toe injuries are minor stretch injuries and require only short periods of treatment. Taping of the hallux to restrict dorsiflexion and/or shoe modifications can be helpful to both allow a return to sport as well as prevent recurrence but should be avoided acutely. Turf toe metal or carbon forefoot plates, Morton’s extensions can be used to alleviate symptoms and prevent recurrence [5].

Grade 2 turf toe injuries are partial disruption injuries. Progression of treatment is similar to grade 1 injuries but requires prolonged treatment at each stage. Early passive range of motion should be followed by protected low-impact activities as symptoms allow. The toe should be protected with a boot or cast immobilization followed by taping as symptoms improve. A turf toe plate or Morton’s extension should be implemented with taping as needed.

Grade 3 turf toe injuries are complete ruptures of the capsuloligamentous complex. Surgical intervention is often required if proximal migration of the sesamoids is evident on imaging.

## The deltoid ligament dilemma: to repair or not to repair?

Deltoid instability is a clearly defined problem in flat foot deformities for which a consensus exists on what and when to treat. For acute deltoid injuries in the athlete, this is not the case as there is a major discussion whether the deltoid should be repaired in acute injuries in the young and athletic population.

Looking at the anatomy of the deltoid ligament, it consists of a deep and superficial portion. The deep portion runs from the talus to the tibia, has a posterior and anterior part, and prevents external rotation of the ankle. The Superficial part is more complex, connecting the tibia with calcaneus, navicular, and spring ligament with the function of blocking hindfoot eversion. We know that not all parts are always present, but the Tibio-navicular, Tibio-spring and deep posterior tibiotalar seem to be present in all so might perhaps be the most important [6]. More importantly, the deltoid seems to play a key role in the stability of the syndesmosis complex and thus a combination of syndesmotic injury and deltoid injury makes it an unstable complex (grade 2b or 3) [7].

The controversy lies with whether or not the deltoid should be repaired in unstable ankle injuries (2b or 3) or in syndesmotic disruptions. Some advocate standard deltoid repair based on their own experience [8], while others remain more conservative because of the lack of evidence [9].

A recent review available on this topic including only 3 prospective studies (1 level 2, 2 level 3 studies) with small sample size, did not prove the superiority of additional medial repair [10]. An important part of this discussion may be the switch from screw fixation to flexible systems. Although the flexible systems might have many benefits, we may progressively introduce the need for deltoid repairs given this preferential implant change [11].

In conclusion, repairing the deltoid in unstable syndesmotic injuries is not yet proven to be beneficial and higher quality research on this topic is needed before we are able to draw definitive conclusions.

## Syndesmosis injuries in athletes: return to play and rehabilitation

Syndesmotic injuries are increasingly common in the field and court sports. Injury severity, nuanced diagnosis, and long-term functional risk have led to significant advances in diagnosis and management protocols. While there is an ongoing need for additional science to support new surgical stabilization constructs and accelerated return to sport protocols, the current management ethos has evolved toward flexible fixation device constructs and more specified rehabilitation protocols.

**Figure 1:**
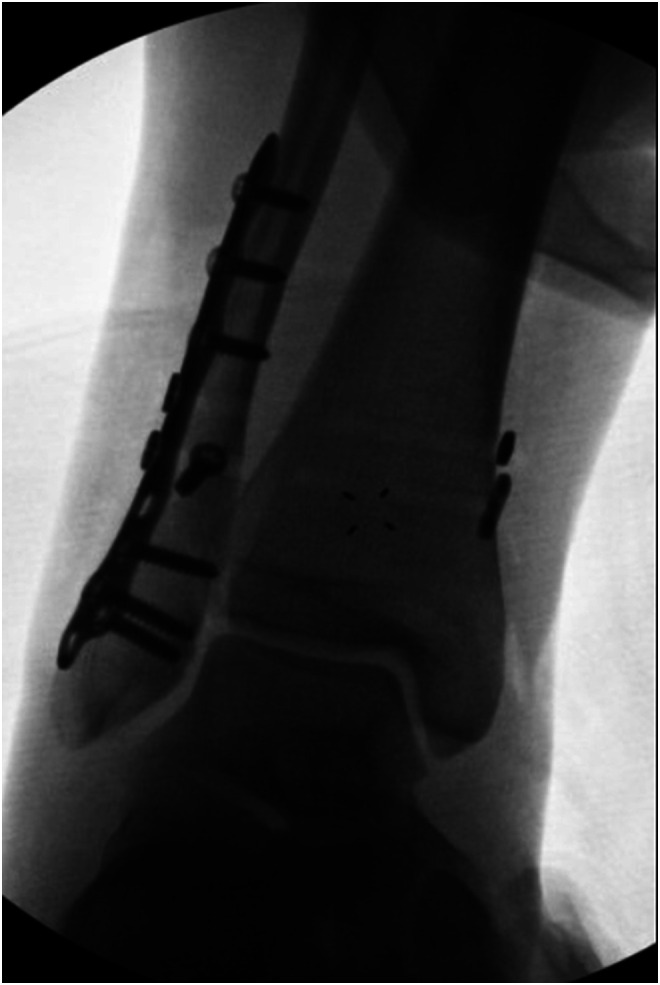
Intra-operative fluoroscopic image of stress test of syndesmosis following fixation of a fibula fracture. Note widening of the syndesmosis and medial clear space, with a lateral shift of the talus relative to the plafond.

The most common clinical examination tests performed to assess syndesmotic injuries include the squeeze test, palpation over the anterior and posterior tibiofibular syndesmosis, lateral translation testing (e.g., the Cotton test), location of tenderness above the ankle joint line, and the fibular instability test. Radiographic imaging, including stress and weight-bearing radiographs (Figure 1), are commonly obtained for treatment decision-making, as are MRI and CT scans of the affected ankle. Magnetic resonance imaging (MRI) is a crucial radiological tool for the assessment of specific ligament injuries in both acute and chronic cases. It is characterized by high sensitivity and specificity and is able to show injuries localized inside the ankle joint, such as bone marrow edema of the calcaneal trochlea, cartilaginous defects, bony injuries, tibiofibular incongruence, or degenerative variations in chronic damages. However, it remains a static exam, which does not provide clear identification of syndesmotic instability. To overcome this problem, physicians need to appeal to functional tests, usually practiced under anesthesia to control the painful feeling around the ankle involved. Management of these injuries is separated into non-operative and surgical protocols based on stable and unstable image findings.

Although screws efficiently stabilize the widened tibiofibular articulation, the technique does not restore a normal biomechanical environment to the syndesmosis joint. In addition, screw breakage and additional surgery for hardware removal are recognized issues. Grassi et al. [12], in their meta-analysis of randomized controlled trials, found that the dynamic fixation of syndesmotic injuries was able to reduce the number of complications and improve clinical outcomes as compared with static screw fixation – especially malreduction and clinical instability or diastasis – at a follow up of 2 years.

**Figure 2:**
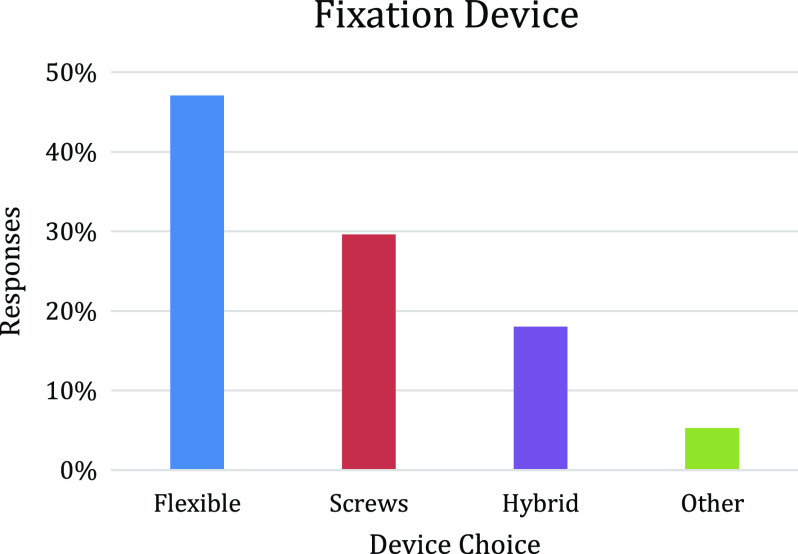
Graph illustrating percentage of surveyed surgeons that would choose one of four techniques (flexible fixation, surgical screws, hybrid construct, or other).

ISAKOS Ankle and Foot committee recently surveyed 742 orthopedic surgeons specializing in ankle injuries from across the globe through ISAKOS and all major orthopedic sports medicine societies. Survey participants answered questions focused on their indications for the treatment of syndesmotic injuries and the information that was used during their decision-making process. Flexible devices were the preferred fixation construct (47.1%), followed by screws (29.6%), hybrid fixation (18%), and other (5.3%) (Figure 2). From our survey collection, we were able to infer that regardless of the severity of the injury to the syndesmosis, device choice and return to play protocol were not consistent internationally although the mean values of survey responses and those in D’Hooghe et al. [13] were strikingly similar.

**Figure 3:**
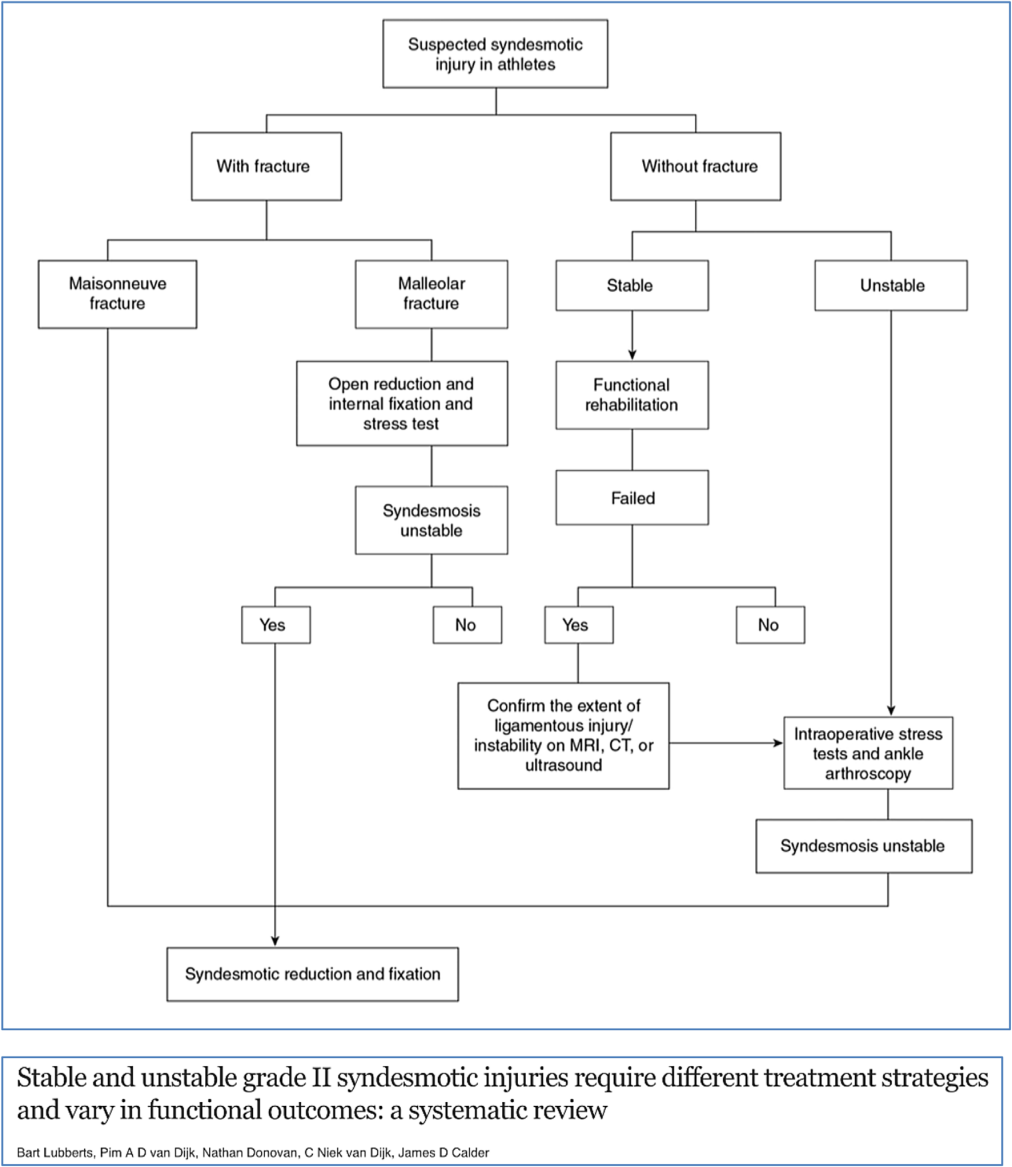
Treatment algorithm for a suspected syndesmotic injury (adapted from Ballal et al. [21]).

Although there are clear differences in etiology, mechanism of injury, and treatment options/RTP between lateral ligament ankle injuries and syndesmotic injuries, most syndesmotic ankle sprains are nowadays still treated by “classic” ankle sprain rehabilitation protocols. New rehabilitation and discharge criteria protocols are necessary with a focus on the specific biomechanics of the ankle syndesmosis.

## Proximal fifth metatarsal fractures

Fracture of the proximal fifth metatarsal was originally described by Jones after an evening of dancing (Jones, 1902 [14]). While past literature contained some confusion about the exact definition of this entity, we define it currently as a fracture of the proximal meta-diaphysis that extends into the fourth-fifth metatarsal articulation or zone 2 as defined by Quill [15] whereas stress fractures are located in the proximal diaphysis (zone 3). Both fracture types are known for poorer healing characteristics. The anatomic region of a Jones and stress fracture acts as a fulcrum between strong ligamentous and tendinous attachments [16, 17] in addition to being a watershed area of blood supply [18], fracture aetiology is thought to reflect both low energy acute and stress mechanisms. Professional basketball players with pes planus and metatarsus adductus have an exaggerated curved 5th metatarsal shaft with a prominent base and are at higher risk of Jones fracture with a 30% nonunion rate [19].

Jones fractures can be difficult to treat with delayed and non-union attributed to the poor blood supply and high mechanical demand. While the evidence is relatively weak (Level 4), studies indicate a higher rate of union with operative treatment at 96% compared to 76% for nonoperative treatment of acute fractures. Delayed unions treated non-operatively healed in 44% while ORIF healed 97% of delayed and nonunions [20]. As the prevalence of refracture remains high in athletes at 4–12% [20], a low threshold for operative treatment is advocated in athletes.

The goals of operative treatment of Jones fractures are to expedite healing, enable rapid rehabilitation, and decrease refracture risk. The authors advocate careful assessment of the foot and ankle alignment using weight-bearing radiographs as part of pre-op planning. Early MRI may help detect a developing stress reaction in high-risk athletes [19]. A mini-open approach is advocated using intramedullary fixation with a solid screw inserted via a high and inside start point to access the canal using cannulated reaming. Using the largest screw diameter that comfortably fits the medullary canal, typically a 5.5 or 6.5 mm solid screw, avoiding headless or cannulated screws. Correction of any alignment deformity may be required concurrently or subsequently.

Failure of operative treatment results from low strength of the fixation, its malpositioning, local biologic factors including poor blood supply, and mechanical alignment. Fixation failure is typically a tension-sided problem with small screw diameter and early vigorous return to activity reported as contributing factors [17]. Low levels of Vitamin D were detected in 65% of Jones fractures [25].

The author’s post-operative protocol includes treatment with a bone stimulator and vitamin D supplementation, 2 weeks in a nonweight bearing splint followed by four weeks in a weight-bearing boot. Running may start at six weeks if the fracture is healed, integrating into the sport at 8–10 weeks. A clamshell insole helps to post the heel laterally and unloads the fifth metatarsal tuberosity enabling an earlier return to activity without high magnitude repetitive loading. Evaluation with CT is advised if there is recurrent or ongoing pain or other risk factors.

Return to sports can be achieved before 10 weeks post-ORIF in 36% of NFL athletes but a revision rate of 60% and decreased sports performance have been reported (public data). On the other hand, NFL players returning to sports more than 10 weeks post-op have a 15% revision rate (public data). Most Jones fractures heal after ORIF but in the event of failure, the contributing factors are identifiable in most cases. Rare complications such as thermal necrosis post intramedullary fixation have been described [26]. The use of bone graft may also be warranted in specific cases when indicated [27]. Close attention to alignment is advocated in all cases with a low threshold for fixation and a tailored post-operative protocol advised in high-level athletes.

## Discussion

Return to Play (RTP) outcomes following numerous orthopaedic injuries and procedures have been documented in a number of professional athletes in the National Basketball Association (NBA), National Football League (NFL), Major League Baseball (MLB), and National Hockey League (NHL) [28–32]. Formal return to play (RTP) criteria have been developed for some orthopedic injuries, most notably anterior cruciate ligament reconstruction, although many of these guidelines are still being developed [33].

Measures to assess RTP are varied and different within published data. These even vary from one injury to another and from one sporting activity to another. RTP assessment might range from simple patient interviews to using sports participation scores or up to assessing objective sports performance indicators [28, 34]. The measures used to assess return to play should be able to address multiple aspects of the sporting activity, such as volume/frequency of play, type of sport, specific sport demands, level of play, and performance parameters. Consequently, the accumulation of all these aspects could provide input on developing RTP measures that will be as comprehensive and sport-specific as possible.

Of the various foot and ankle assessment scales, the Tegner score [35] and The Foot and Ankle Outcome Score [36], and Foot and Ankle Ability Measure [37] have sport-specific scales. These scoring systems might point to certain aspects of RTP however, they were not developed specifically for use in an exclusively athletic population and many have not been validated specifically for certain injuries or indeed for certain specific sports.

Even regarding the mere definition of a time frame for RTP, various perspectives have been proposed. In a cohort of NFL players, Yang and co-workers defined RTP as the first regular-season or postseason game in which the athlete played after injury. Play in a preseason game was used as the date of RTP only if the athlete subsequently played in an RS or playoff game during that same season [38].

On the other hand; Grassi et al. [12] when looking at professional soccer players regarding Achilles tendon injuries defined the time to return to competition as the time from injury to the participation of at least 1 min of an official match. They also defined return to the previous level of play as a player playing for at least two entire seasons after the return to unrestricted training following ATR and at least five matches per season in the same division as before he suffered the index injury.

Recovery progression after an injury or surgery in an elite athlete may be different from that of a recreational sports person. One might assume that, because of the very high levels of performance expected, the elite athlete may take longer to return to a preinjury level than the average person. However, better access to expert aftercare by dedicated physical therapists and issues of motivation often mean that a high-performance athlete rehabilitates more quickly [39].

Regarding the specific injuries being currently discussed. In Lisfranc injuries, although there are limited studies in the athletic population, they show that a majority of patients are able to preinjury activities including elite sports regardless of treatment method (ORIF vs. Fusion) [40–43].

On the other hand, regarding deltoid injuries occurring in combination with unstable syndesmotic injuries (Stage 2B or 3 injuries), it has been shown in large series of athletes, that the result of only treating the syndesmosis in athletes the outcome and return to sports is good, so the question remains as to what is there that can be improved [44].

Similarly, there is no current consensus on a return to sport protocol for athletes to return to play following a syndesmotic injury. Data from a large study group [13] revealed that the mean time to begin on-field/sport-specific rehabilitation was 37 ± 12 days, with a mean time of 103 ± 28 days to the first match after syndesmotic stabilization [13].

## Conclusion

The foot and ankle complex are the most common area of orthopaedic injury in sports. While the severity of the commonest injuries discussed here may vary from simple sprains with early recovery to injuries at the severe end of the spectrum, an accurate diagnosis and early treatment are paramount. It is the responsibility of the treating clinician to be cognizant of classic symptoms and subtle signs of significant injuries that will ultimately shape the prognosis and time to return to sport.

## Conflicts of interest

The authors declare that they have no conflicts of interest in relation to this article.
